# Smad8 Is Increased in Duchenne Muscular Dystrophy and Suppresses miR-1, miR-133a, and miR-133b

**DOI:** 10.3390/ijms23147515

**Published:** 2022-07-07

**Authors:** Michael A. Lopez, Ying Si, Xianzhen Hu, Valentyna Williams, Fuad Qushair, Jackson Carlyle, Lyndsy Alesce, Michael Conklin, Shawn Gilbert, Marcas M. Bamman, Matthew S. Alexander, Peter H. King

**Affiliations:** 1 Children’s of Alabama, Birmingham, AL 35233, USA; mconklin@uabmc.edu (M.C.); srgilbert@uabmc.edu (S.G.); matthewalexander@uabmc.edu (M.S.A.); 2 Department of Pediatrics, University of Alabama at Birmingham (UAB), CHB314, 1600 7th Avenue South, Birmingham, AL 35233, USA; xianhu@uab.edu (X.H.); vwilliams@auburn.vcom.edu (V.W.); fuadq@uab.edu (F.Q.); jcarlyle@auburn.vcom.edu (J.C.); 3 Department of Neurology, University of Alabama at Birmingham (UAB), Civitan 545C, 1530 3rd Avenue South, Birmingham, AL 35294, USA; yingsi@uabmc.edu (Y.S.); lalesce@uabmc.edu (L.A.); mbamman@ihmc.org (M.M.B.); 4 UAB Center for Exercise Medicine (UCEM), University of Alabama at Birmingham (UAB), Birmingham, AL 35233, USA; 5 Birmingham Veterans Affairs Medical Center, Birmingham, AL 35233, USA; 6 Department of Orthopedic Surgery, University of Alabama at Birmingham (UAB), Birmingham, AL 35233, USA; 7 Department of Cell, Development and Integrative Biology, Birmingham, AL 35233, USA; 8 UAB Civitan International Research Center (CIRC), Birmingham, AL 35233, USA; 9 Department of Genetics, University of Alabama at Birmingham (UAB), Birmingham, AL 35233, USA

**Keywords:** BMP4, Duchenne, miRNA, muscle, Smad8

## Abstract

Duchenne muscular dystrophy (DMD) is an X-linked recessive disease characterized by skeletal muscle instability, progressive muscle wasting, and fibrosis. A major driver of DMD pathology stems from aberrant upregulation of transforming growth factor β (TGFβ) signaling. In this report, we investigated the major transducers of TGFβ signaling, i.e., receptor Smads (R-Smads), in DMD patient skeletal muscle and observed a 48-fold increase in *Smad8* mRNA. *Smad1*, *Smad2*, *Smad3*, and *Smad5* mRNA were only minimally increased. A similar pattern was observed in the muscle from the *mdx^5cv^* mouse. Western blot analysis showed upregulation of phosphorylated Smad1, Smad5, and Smad8 compared to total Smad indicating activation of this pathway. In parallel, we observed a profound diminishment of muscle-enriched microRNAs (myomiRs): miR-1, miR-133a, and miR-133b. The pattern of Smad8 induction and myomiR suppression was recapitulated in C2C12 muscle cells after stimulation with bone morphogenetic protein 4 (BMP4), a signaling factor that we found upregulated in DMD muscle. Silencing *Smad8* in C2C12 myoblasts derepressed myomiRs and promoted myoblast differentiation; there was also a concomitant upregulation of myogenic regulatory factors (myogenin and myocyte enhancer factor 2D) and suppression of a pro-inflammatory cytokine (interleukin-6). Our data suggest that Smad8 is a negative regulator of miR-1, miR-133a, and miR-133b in muscle cells and that the BMP4-Smad8 axis is a driver of dystrophic pathology in DMD.

## 1. Introduction

Duchenne muscular dystrophy (DMD) is an X-linked recessive skeletal muscle wasting disease. Worldwide, it affects 1 out of every 3500 to 6000 newborn males [1,2,3]. Loss of dystrophin protein destabilizes the sarcolemmal membrane and leads to chronic muscle degeneration, regeneration, inflammation, and fibrosis [4,5]. Sarcolemmal membrane instability leaves skeletal muscle susceptible to stress-induced mechanical injury; this is followed by a chronic asynchronous process of regeneration and degeneration culminating in the exhaustion of regenerative properties of skeletal muscle [6]. A myriad of molecular pathways are involved, but key amongst them are the immune-mediated pathways that contribute to clinical progression [7,8]. 

The transforming growth factor beta (TGFβ) superfamily of receptors transduces key immune signals and has pleiotropic cellular effects [9]. The TGFβ superfamily receptor ligands are numerous and broadly include families of secreted cytokines such as TGFβ and bone morphogenetic proteins (BMP); these cytokines activate specific hetero-tetrameric combinations of type I and type II receptors, i.e., TGFβ type I (R1), TGFβ type II (R2), BMP type I, and BMP type II receptors. Upon type I receptor activation, serine/threonine kinases phosphorylate the receptor Smads (R-Smads) which include Smad1, Smad2, Smad3, Smad5, and Smad8. Phosphorylated R-Smads then transduce the signal by binding to Smad4 to translocate into the nucleus. Classically, two canonical signaling transduction pathways are known to include the TGFβ R1/R2 for Smad2 and Smad3 (Smad2/3) and BMP type I/II receptors for Smad1, Smad5, and Smad8 (Smad1/5/8). The TGFβ-Smad2/3 signaling induces muscle atrophy and promotes fibrosis, whereas its inhibition leads to muscle hypertrophy [10,11,12,13,14,15]. In contrast, the BMP-Smad1/5/8 pathway is important in hypertrophic signaling [15]. Together, the R-Smads are integral to skeletal muscle health and disease [12,16,17].

In DMD, TGFβ1 is a well-described modifier of disease; it promotes end-stage muscle disease typified by fibro-adipocytic replacement of skeletal muscle [12,18]. TGFβ1 is increased in DMD and positively correlates with disease severity [19,20,21,22]. Several clinical trials have tested drugs that inhibit myostatin, an activator of the TGFβ-Smad2/3 pathway, in an attempt to mitigate muscle atrophy at the receptor level (NCT02515669, NCT02310763) [23]; however, the clinical efficacy of anti-myostatin drugs has not been shown; this underscores the complexity of dystrophic signaling in DMD. Unlike TGFβ1, BMP4 is not well-studied, but is also increased in DMD and is associated with impaired muscle regeneration [24]. BMP4 is part of a group of secreted BMPs that have pro-osteogenic properties [25]. Antagonism of BMP4 ameliorates fibrosis and increases myogenic markers in an mdx mouse model [26]. The BMP-Smad1/5/8 pathway, however, is not well characterized in the context of DMD pathophysiology, especially Smad8, which is a known regulator of microRNAs (miRNA). 

miRNAs are epigenetic regulators of genetic programs, and their loss is associated with dystrophic disease [27,28,29]. For example, we have shown that miR-486 is aberrantly decreased in DMD and restoration of miR-486 expression ameliorates dystrophic muscle pathology in the *mdx^5cv^* mouse [30]. Other studies have shown that deletion of miR-133b worsens dystrophic DMD pathology through impairment of muscle biogenesis and enhancement of pro-inflammatory (e.g., STAT3/IL-6) and pro-fibrotic signaling [31,32]. 

Our interest in Smad8 emerged from our earlier studies of patients with amyotrophic lateral sclerosis (ALS). We identified Smad8 as a muscle biomarker that increases with disease progression in parallel with increased TGFβ and decreased muscle-enriched microRNAs (myomiRs): miR-1, miR-133a, and miR-133b [16,33,34]. Although primarily a neurogenic disease, ALS muscle pathology shares several features with DMD including muscle atrophy, fibrosis, inflammation, and increased TGFβ signaling [35,36,37,38,39]; this prompted us to assess R-Smads and their impact on myomiRs in human DMD and *mdx^5cv^* mouse skeletal muscles. We hypothesized that Smad8 would be increased in DMD skeletal muscle and activated through BMP4 to promote dysregulation of miRNAs. 

## 2. Results

### 2.1. Smad8 Expression Is Increased in Human DMD and mdx^5cv^ Skeletal Muscles

**Figure 1:**
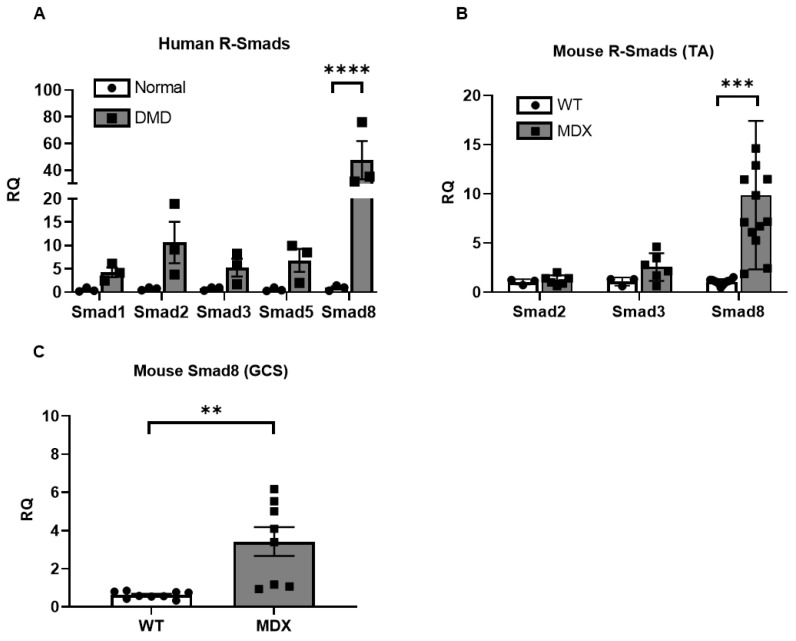
*Smad8* mRNA is selectively upregulated in DMD and *mdx^5cv^* (MDX) skeletal muscles. (**A**) *Smad8* mRNA expression levels, determined by qPCR, show a significant increase in human DMD versus healthy control muscle (Normal). *Smad8* mRNA levels were also increased in the tibialis anterior (TA) muscle (**B**) and gastrocnemius (GCS) muscle (**C**) in MDX versus wild-type mice at 6 months of age. Data points show biological replicates for each group. Bars show mean ± SEM. *p* values: ** < 0.01, *** < 0.001, and **** < 0.0001. RQ, relative quantity.

Quantitative PCR (qPCR) analysis of DMD skeletal muscles showed *Smad8* mRNA levels increased by ~48-fold over normal muscles (*p* < 0.0001; Figure 1A). Other R-Smads trended higher but did not reach statistical significance; this pattern was similar in the *mdx^5cv^* mouse, a model of severe DMD. At 6 months old, *Smad8* was increased in the tibialis anterior muscle by 9.8-fold, but *Smad2* and *Smad3* were comparable to wildtype (Figure 1B). Similarly, *Smad8* was increased in *mdx^5cv^* gastrocnemius muscle by nearly 3.4-fold over wildtype (Figure 1C). 

**Figure 2:**
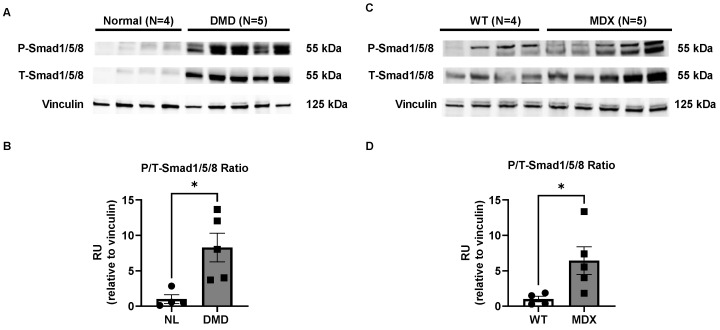
Smad1/5/8 total (T-) and phosphorylated (P-) protein is increased in dystrophic skeletal muscles. (**A**) Human DMD muscle shows increased T-Smad1/5/8 and P-Smad1/5/8 by western blot. (**B**) Densitometric analysis showed an increased fraction of P-Smad1/5/8 compared with T-Smad1/5/8 (P/T-Smad1/5/8 ratio). (**C**,**D**) 9-month-old *mdx^5cv^* tibialis anterior muscle showed a similar pattern of increased P/T-Smad1/5/8 ratio. Vinculin is shown for comparison of loading. Data points show biological replicates for each group. Bars show mean ± SEM. *p*-value: * < 0.05. RU, relative units.

We next assessed Smad8 by western blot. We used an antibody that derepressive detects Smad1, 5, and 8 (total Smad1/5/8) because there is no specific antibody to Smad8 due to the amino acid conservation between Smad1, 5, and 8 [34]. There was a marked increase in total (T-) and phosphorylated (P-) Smad1/5/8 in human DMD muscle compared with controls (Figure 2A). Quantitative densitometry showed an 8-fold increase in the ratio of P-Smad1/5/8 relative to T-Smad1/5/8 (P/T-Smad1/5/8) in DMD muscles (Figure 2B). The loading control, vinculin, was unchanged. The pattern of increased P/T-Smad1/5/8 ratio was reproduced in 9-month-old *mdx^5cv^* TA muscles (Figure 2C,D). 6-month-old *mdx^5cv^* TA muscles did not show a significant difference in the P/T-Smad1/5/8 ratio (Figure S1). Taken together, Smad8 activation is increased in DMD and appears to be a biomarker of disease progression in the *mdx^5cv^* model.

### 2.2. miR-1, miR-133a, and miR-133b Are Repressed in Human DMD and mdx^5cv^ Skeletal Muscles

**Figure 3:**
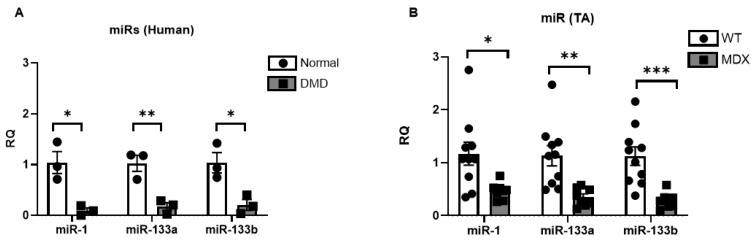
miR-1, miR-133a, and miR-133b are repressed in DMD and *mdx^5cv^* (MDX) skeletal muscles. (**A**) DMD human skeletal muscles show reduced miRNA expression levels by qPCR. (**B**) 6-month-old *mdx^5cv^* (MDX) mouse tibialis anterior muscles show reduced miR-1, miR-133a, and miR-133b compared with wildtype (WT). Data points show biological replicates for each group. Bars show mean ± SEM. *p* values: * < 0.05, ** < 0.01, and *** < 0.001. RQ, relative quantity.

Our prior work in ALS muscle revealed an inverse relationship between Smad8 levels (increased) and myomiRs (decreased; miR-1, miR-133a, and miR-133b) [33,34]. Here, we sought to determine whether a similar pattern was present in DMD. Using human DMD muscle samples, we observed even larger reductions of miR-1 (95%), miR-133a (85%), and miR-133b (83%) in dystrophic skeletal muscles (Figure 3A). In the 6-month-old *mdx^5cv^* mouse TA, the mean fold-reduction of myomiRs ranged from 71% to 84% at 6 months old (Figure 3B). These data indicate that the myomiRs are diminished in DMD muscle inversely to increased expression of Smad8.

### 2.3. Silencing Smad8 Increases miR-1, miR-133a, and miR-133b

**Figure 4:**
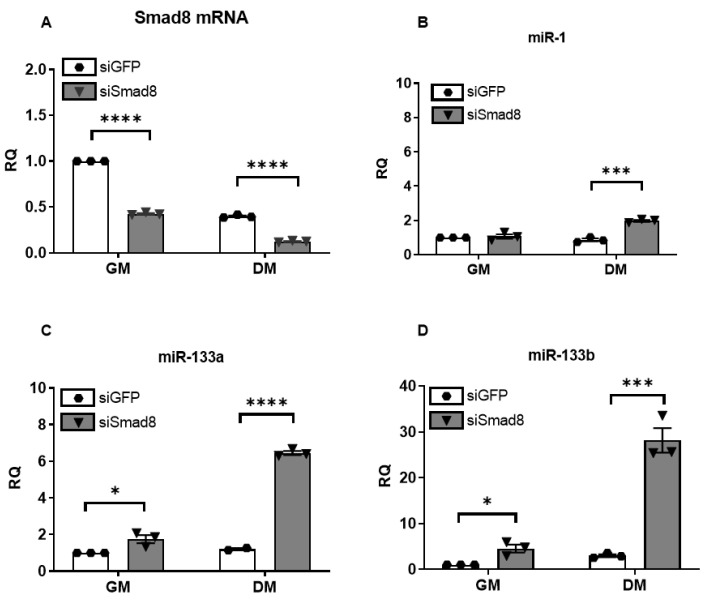
*Smad8* silencing in C2C12 cells increases miR-1, miR-133a, and miR-133b expression by qPCR. (**A**) *Smad8* silencing (siSmad8) achieved >50% reduction of *Smad8* mRNA compared with siRNA control (siGFP) in growth (GM) and differentiation media (DM). (**B**–**D**) miR-1, miR-133a, and miR-133b increased after *Smad8* knockdown. Data points show biological replicates for each group. Bars show ± SEM. *p* values: * < 0.05, *** < 0.001, and **** < 0.0001. RQ, relative quantity.

**Figure 5:**
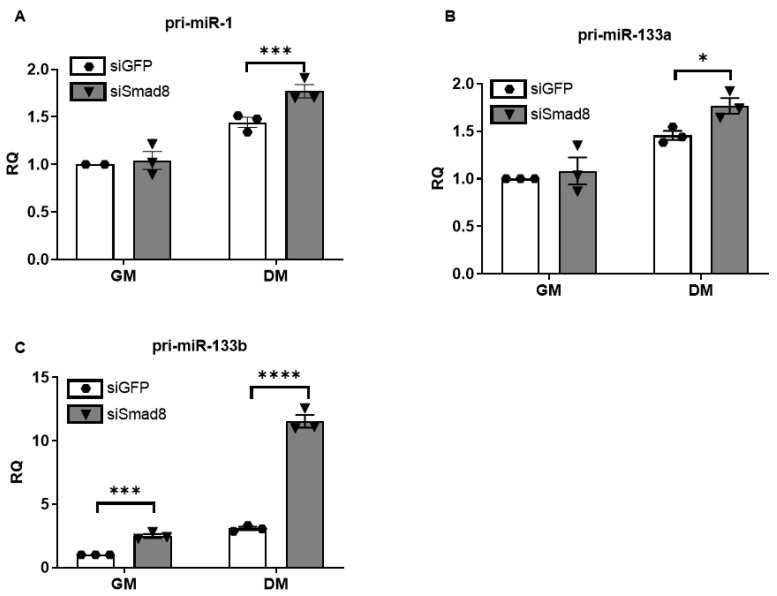
Primary myomiR transcripts are increased after *Smad8* silencing (siSmad8) in C2C12 cells by qPCR. (**A**–**C**) Primary miRNA (pri-miR) levels for pri-miR-1, pri-miR-133a, and pri-miR-133b are increased in siSmad8- vs. siControl- (siGFP) treated cells in growth (GM) and differentiation media (DM). Data points show biological replicates for each group. Bars show ± SEM. *p* values: * < 0.05, *** < 0.001, and **** < 0.0001. RQ, relative quantity.

We utilized siRNA to knock down *Smad8* mRNA expression in C2C12 mouse myoblasts to explore the relationship between *Smad8* and repressed myomiRs. After transfection with siSmad8, we achieved more than 50% reduction of *Smad8* mRNA (Figure 4A). *Smad8* levels decreased under differentiation medium (DM) consistent with a prior report [40]. There was a marked increase in miR-1, miR-133a, and miR-133b levels compared with the siRNA control (Figure 4B–D). The increase in miRNAs was smaller in growth medium (GM) compared with DM, but still significant for miR-133a and miR-133b (Figure 4C–D). miR-133b showed the largest increase at 28-fold in DM. Primary miRNA transcript 133b (pri-miR-133b) showed a maximum ~11.5-fold increase after *Smad8* silencing (Figure 5), whereas pri-miR-1 and pri-miR-133a showed smaller but still significant increases. The concomitant increase in pri-miRNAs suggests a transcriptional effect, especially for miR-133b. 

### 2.4. BMP4 Stimulation Induces Smad8 Expression in C2C12 Cells and Suppresses miR-1, miR-133a, and miR-133b

**Figure 6:**
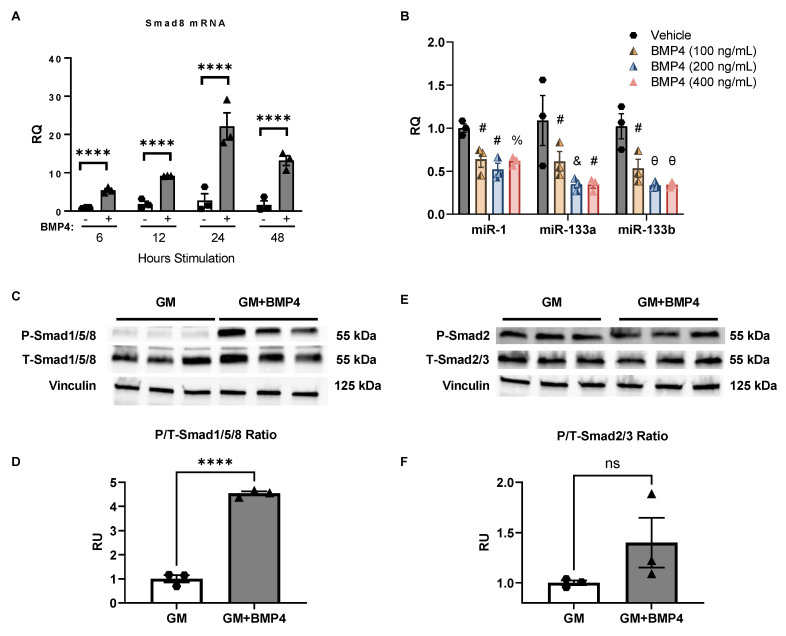
BMP4 induces activated *Smad8* in C2C12 myoblasts and suppresses myomiRs. (**A**) *Smad8* mRNA levels were quantitated by qPCR in C2C12 cells after stimulation with BMP4 for different durations in growth media (GM). *Smad8* mRNA induction peaked at 24 hours. (**B**) qPCR results show a dose-dependent repressive effect on miR-1, miR-133a, and miR-133b at 24 h. (**C**,**D**) Western blot of C2C12 cells stimulated with BMP4 (300 ng/mL for 24 h) shows induction of P-Smad1/5/8 with a greater than 4-fold increase in P/T-Smad1/5/8 ratio by densitometry. (**E**,**F**) Western blot and densitometry of total Smad2/3 (T-Smad2/3) and phosphorylated Smad2 (P-Smad2) show no significant induction in BMP4-stimulated cells. Bars show mean ± SEM of 3 biological replicates per group. *p* values (compared to vehicle): % < 0.05, # < 0.01, & < 0.001, **** < 0.0001, and θ < 0.0001. RQ, relative quantity. RU, relative units.

To begin assessing signaling pathways that might be driving Smad8 upregulation in DMD, we focused on BMP4, which classically activates Smad1/5/8 through BMP receptors and is upregulated in DMD [15,24,41]. We stimulated C2C12 cells with BMP4 recombinant protein for varying durations and concentrations in GM (Figure 6A,B). At 24 h, *Smad8* mRNA increased by 22-fold over vehicle-treated cells (Figure 6A). Using the time point of 24 h, we observed a dose-dependent suppression of myomiRs by BMP4 (Figure 6B). At 400 ng/mL, there was a 70% reduction for miR-133a and miR-133b and 31% reduction for miR-1. 

We next assessed the phosphorylation status of the Smad1/5/8 and Smad2/3 in BMP4-stimulated C2C12 cells. There was a ~4.5-fold increase in the P/T-Smad1/5/8 ratio after 24 h (Figure 6C,D). In contrast, neither T-Smad2/3 nor P-Smad2 showed differences compared with untreated cells. These data suggest that the BMP4/Smad8 axis is a potential driver of suppression of miR-1, miR-133a, and miR-133b. We next explored if *Smad8* silencing affected key genes involved in DMD signaling. 

### 2.5. Smad8 Silencing in C2C12 Cells Alters Downstream mRNAs Linked to Dystrophic Signaling

**Figure 7:**
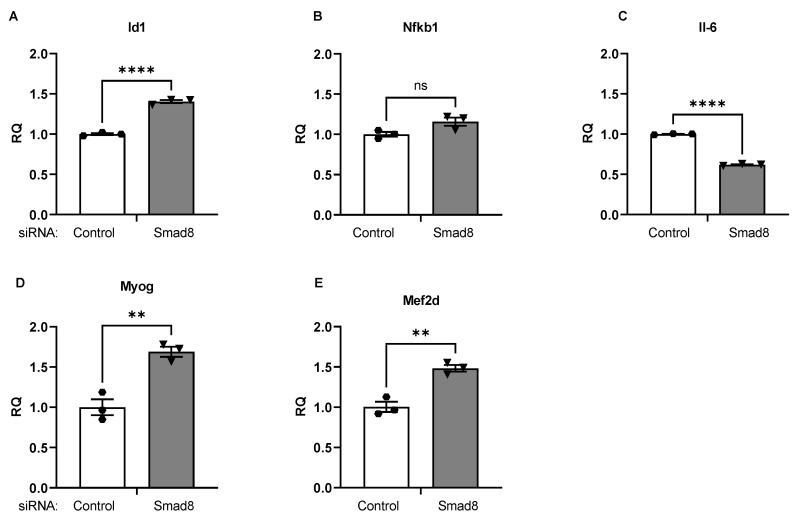
*Smad8* silencing promotes DMD-mitigating mRNA patterns in C2C12 cells. After transfection of siSmad8 or siControl (siGFP), qPCR was used to assess mRNA expression levels of (**A**) *Id1*, a known transcriptional target of *Smad8*, (**B**) *Nfkb1*, (**C**) *Il-6*, a pro-inflammatory cytokine, (**D**) *Myog*, muscle regulatory factor, and (**E**) *Mef2d*, muscle regulatory factor. Bars show mean ± SEM of 3 biological replicates per group. *p* values: ** < 0.01, **** < 0.0001. RQ, relative quantity.

After transfection of C2C12 cells with siRNA targeting *Smad8* (siSmad8), we assayed several downstream mRNA targets relevant to Smad8 signaling and DMD (Figure 7). We observed an increase in *Id1* mRNA, an established BMP-Smad8-regulated target, consistent with prior reports (Figure 7A) [42,43,44]. The mRNA of the pro-inflammatory cytokine *IL-6* was suppressed by ~38%; while *Nfkb1* mRNA was unaffected (Figure 7B,C). We next assessed two pro-myogenic transcription factors, myocyte enhancer function 2d (*MEF2D*) and myogenin (*MYOG*). *MEF2* has previously been linked to miR-1 and miR-133 regulation [45]. Both *MEF2D* and *MYOG* mRNAs were significantly increased after *Smad8* silencing (Figure 7D,E).

### 2.6. BMP4/Smad8 Pathway Is Upregulated in DMD Skeletal Muscle

**Figure 8:**
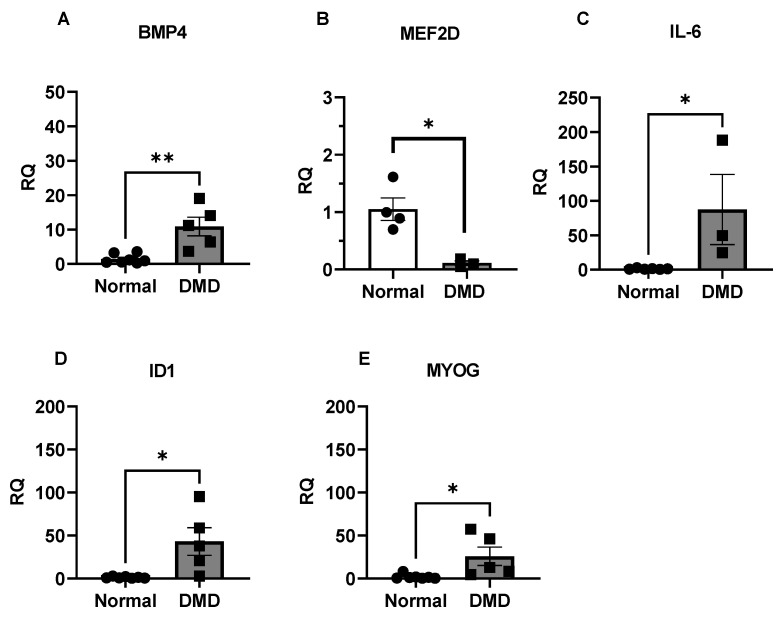
BMP4/Smad8 targets are dysregulated in human DMD skeletal muscles. (**A**–**E**) Results of qPCR analysis of human DMD and normal muscle for different mRNA targets. Data points show biological replicates for each group. Bars show mean ± SEM. *p* values: * < 0.05 and ** < 0.01. RQ, relative quantity.

We next assayed selected mRNA targets in human DMD muscles that were altered by silencing Smad8 or part of the BMP4/Smad8 signaling pathway. We found that *BMP4*, *IL-6*, *ID1*, and *MYOG* mRNAs were all increased in DMD (Figure 8). *BMP4* mRNA was increased by 11-fold (Figure 8A). In contrast, *MEF2D* mRNA was reduced by 9-fold, whereas *IL-6* was increased by ~87-fold (Figure 8B,C). *ID1* and *MYOG* mRNAs were also increased, but to a lesser extent (Figure 8D,E).

### 2.7. Smad8 Silencing in C2C12 Cells Promotes Myogenic Differentiation

**Figure 9:**
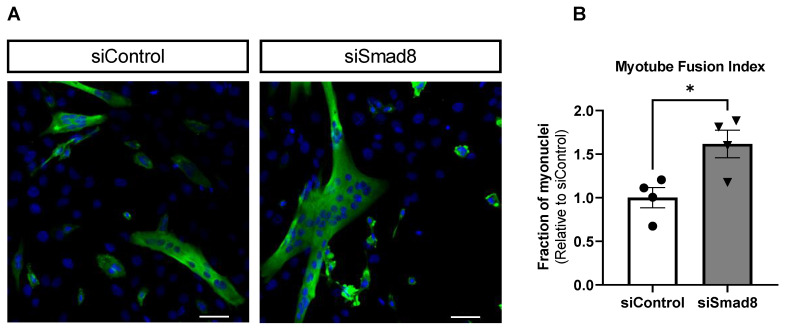
Myotube fusion index is increased after *Smad8* silencing (siSmad8). (**A**) Representative immunocytofluorescence imaging shows C2C12 cells at day 4 of differentiation after transfection of siSmad8 or siControl (siGFP). Cells were stained for MF20 (Green) to identify myotubes and 4′,6-diamidino-2-phenylindole (Blue) for myonuclei. Scale bar = 50 μm. (**B**) Myotube fusion index was increased significantly relative to siControl (* *p* < 0.05).

Silencing *Smad8* increased the C2C12 myotube fusion index by 1.6-fold at day 4 of differentiation (Figure 9). qPCR confirmed *Smad8* mRNA knockdown by 59% compared to siControl (data not shown). These data suggest that *Smad8* inhibition promotes myogenic differentiation.

## 3. Discussion

In this report, we show that *Smad8* is upregulated in skeletal muscle of DMD patients and the DMD mouse model, *mdx^5cv^*, in association with a marked suppression of myomiRs: miR-1, miR-133a, and miR-133b. We show in cultured muscle cells that BMP4 stimulation recapitulates this pattern and *Smad8* silencing rescues myomiR expression. A disease-mitigating effect of *Smad8* silencing is further suggested by the altered downstream mRNA targets including attenuation of the pro-inflammatory cytokine, *IL-6*, and upregulation of pro-myogenic factors *MYOG* and *MEF2D*. *Smad8* silencing also promoted myogenic differentiation.

Smad8 is typically associated with Smad1 and Smad5 as they are phosphorylated (activated) by type I and type II BMP receptors (BMPR) [46]. We observed increased phosphorylation of Smad1/5/8 in DMD muscle consistent with BMPR activation. With the concomitant and dramatic increase in *Smad8* mRNA in DMD muscle, it is likely that these phosphorylated forms represent activated Smad8. This pattern was reproduced in *mdx^5cv^* muscles, including fast- (TA) and slow-twitch muscle groups (GCS) at 9 months old, and this is an age where dystrophic pathology is severe. Canonical Smad signaling via Smad1/5/8 activation is associated with increased myofiber hypertrophy and in DMD muscle may reflect a compensatory mechanism for acute degenerating skeletal muscle. We and others have observed this in the recovery phase after sciatic nerve injury [15,34]. In DMD, sustained hyperactivation of Smad8 over time could dampen the beneficial effects of these compensatory changes and mediate aberrant signaling, such as through myomiR repression. This could then lead to more widespread dysregulation of myogenic pathways targeted by these myomiRs, such as proliferation and differentiation. This may also apply to ALS, a chronic muscle wasting disease of neurogenic origin, where sustained Smad1/5/8 signaling and myomiR suppression are likewise observed [16,34]. 

Our data also demonstrate that BMP4 is upregulated in DMD muscles. Many studies have demonstrated the importance of TGFβ1 in DMD, but comparatively little is known about BMP4’s role. BMP4 is a common ligand for BMPR activation, and we and others have now shown it to be upregulated in DMD muscles and linked with the dystrophic phenotype [24,26]. Our finding that BMP4 stimulation of myoblasts recapitulated *Smad8* induction and myomiR suppression, as observed in human DMD muscle, suggests that the BMP4/Smad8 axis is a driver of dystrophic signaling. Several lines of evidence support this possibility. First, myomiRs are participants in muscle homeostasis and their suppression would impede muscle regeneration in DMD. For example, miR-1 and miR-133 have been implicated in muscle functions such as regulating inflammation, myogenesis, fiber-type switching, and muscle mass [31,47,48]. The second line of evidence emerges from the analysis of downstream mRNAs regulated by Smad8. *Id1* was increased after *Smad8* silencing and elevated in DMD muscle. Id1 inhibits basic helix loop helix (BHLH) DNA binding, mediates BMP signaling, and is increased after muscle injury. It has been postulated to promote expansion of myoblasts necessary for muscle regeneration [49]; however, *Id1* is also a negative regulator of myoblast differentiation. As with other myogenic regulatory factors, it may be necessary for proliferation and regeneration, but then reduced to allow for myoblast differentiation. This would suggest that *Smad8* silencing would favor myoblast differentiation over-proliferation. Indeed, we found that *Smad8* silencing reversed the suppression of pro-myogenic factors *MYOG* and *MEF2D* and promoted myoblast differentiation. MYOG and MEF2D play important roles in myogenesis, prevention of atrophy, and are part of a family muscle regulatory factors (MYOD, MEF2, MYF5, MYF6, and SRF) that additionally promote myomiR biogenesis [50,51]. In contrast, *IL-6* mRNA was attenuated with *Smad8* silencing. IL-6 is a pro-inflammatory cytokine that exacerbates the dystrophic process by promoting the recruitment of neutrophils and other immune cells to sustain an inflammatory milieu [52,53]. This impedes muscle regeneration and promotes fibrosis. 

In parallel with these mRNA changes, we showed that miR-1, miR-133a, miR-133b are repressed in human DMD muscle and *mdx^5cv^* skeletal muscles. These findings are similar to other reports demonstrating repression of miR-1 and miR-486 in DMD muscle [27,29]. A known function of Smads is to modulate the biosynthesis of miRNAs directly through binding to Smad Binding Elements (SBE) in the promoter of miRNAs, indirectly through the regulation of other miRNA transcription factors, or through the maturation of miRNAs post-transcriptionally [46,54]. Our finding that primary myomiR transcripts increased with *Smad8* silencing is supportive of regulation at the transcriptional level. Although for miR-1 and miR-133a, post-transcriptional miRNA maturation might be more important because of the modest derepressive effect of *Smad8* silencing on the primary transcripts. The transcriptional regulation of myomiRs is found in several clusters including separate gene clusters for miR-1/miR-133a and miR-206/miR-133b which may facilitate redundancy via independent transcriptional mechanisms [55]. 

**Figure 10:**
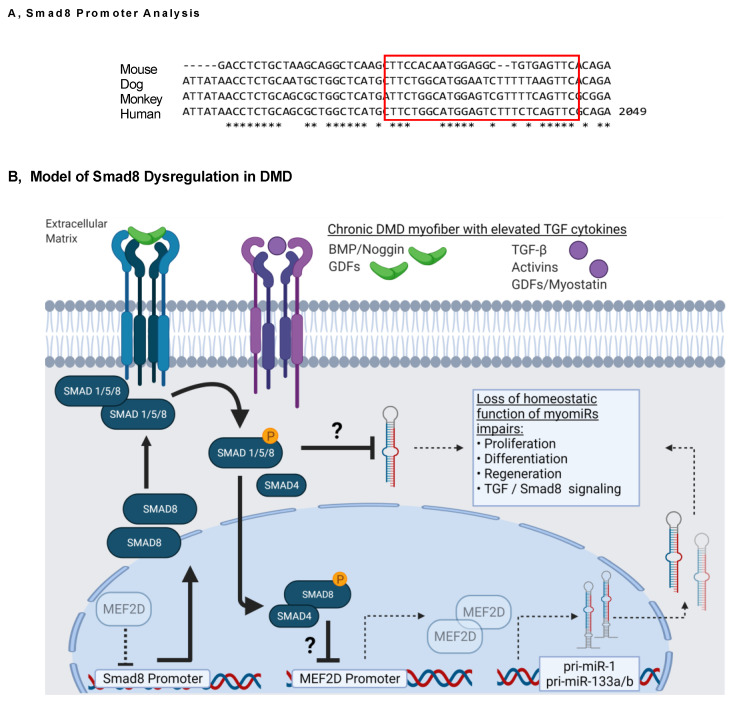
Proposed model of *Smad8* repression of myomiRs. (**A**) The *SMAD8* promoter (+500 to −2 kb from the start site) contains a highly conserved *MEF2* transcription factor binding site (red box). (**B**) Our model proposes that chronic upregulation of TGF receptor ligands in DMD leads to the induction and sustained activation of Smad8, which inhibits the *MEF2D* promoter and represses MEF2D-mediated myomiR transcription; this results in loss of myomiR homeostatic functions. An alternate post-transcriptional effect of Smad8 on mature myomiR expression is also possible. TGFβ1-Smad2/3 is not shown for simplicity. The model was generated after a review of the literature [9,42,54,59].

Indeed, MEF2 activates transcription of primary miR-1 and miR-133 [45,56]. We found *MEF2D* mRNA to be suppressed in human DMD muscle and restored with *Smad8* silencing. Others have found that the expression of myogenic regulatory factors (MRF), i.e., MYOG, MYOD, and MEF2D, are inhibited by TGFβ and that the R-Smad inhibitor, Smad7, reverses this inhibition when ectopically expressed [57,58]. Additionally, *MEF2D* transcriptional regulation has been reported to be suppressed by myostatin-mediated Smad2/3 activation [14]. A regulatory connection between *Smad8* and *MEF2* is further supported by the presence of a highly conserved SBE within the promoter of *MEF2* (Figure 10A). This suggests a potential mechanism for Smad8 suppression of miR-1 and miR-133a/b via transcriptional inhibition of *MEF2D* by Smad8, which blocks the negative feedback effect of MEF2D on the Smad8 promoter (Figure 10B). *MEF2D* suppression then leads to attenuated myomiR transcription and subsequent dysregulation of homeostatic skeletal muscle functions. 

## 4. Methods

### 4.1. Human Muscle Samples

Human muscle biopsies were collected from DMD patients under an approved Institutional Review Board protocol (IRB00000196). For DMD muscle biopsies, a small portion of erector spinae or multifidus muscle was biopsied using a typical posterior midline approach to the thoracolumbar spine. Human control muscle samples were selected from the archive of remnant muscle biopsy tissues, with normal histopathological diagnosis, at the UAB Division of Neuromuscular Disease as previously detailed [34]. The control patients ranged in age from 16 to 33 years old. DMD male patients ranged in age from 12 to 20 years old.

### 4.2. Mouse Muscle Samples

The *mdx^5cv^* mouse strain (#002379) and wildtype C57BL/6J strain (#000664) were obtained from Jackson Laboratories. All mouse strains were approved for experimental studies by the UAB Institutional Animal Care and Use Committee (IACUC) (Protocol #21393). All mice were housed in pathogen-free, sterile conditions with standard housing and feedings set by the University of Alabama at Birmingham Animal Resources Facility. Six-month-old male mice were used in all experimental conditions except when noted otherwise. 

### 4.3. Cell Culture and Transfection

C2C12 cells were grown in growth or differentiation media as noted. 6-well plates were seeded with 0.3 MM cells per well 24 h prior to transfection. Transfections of siSmad8 (Horizon Discovery Biosciences Limited, Cambridge, UK; Ref# Smad9 Mouse On-Target Plus siRNA) were done at ~80% confluency using Lipofectamine 2000 (Invitrogen/Thermo Fisher Scientific, Waltham, MA, USA). siGFP (Horizon Discovery Biosciences Limited, Cambridge, UK) was used as an siRNA control transfection. Cells were treated with BMP4 recombinant protein (R&D System/Thermo Fisher Scientific, Waltham, MA, USA; Ref# 5020-BP) at specified concentrations. RNA or protein were collected using miRVana Kit or M-PER lysis buffer, respectively. 

### 4.4. RNA Isolation and qPCR Analysis

Total RNA and miRNA were isolated from muscle tissue using the miRVana Isolation Kit (Thermo Fisher Scientific, Waltham, MA, USA; Ref# AM1560) following the manufacturer’s procedure. cDNA was synthesized using SuperScript™ IV First-Strand Synthesis System (Invitrogen/Thermo Fisher Scientific, Waltham, MA, USA) using 1 ug of RNA. Taqman assay probes and primers (Applied Biosystems/Thermo Fisher Scientific, Waltham, MA, USA). Taqman probes used for qPCR experiments are listed in Supplemental Table S1 and S2. TaqMan reactions were performed using TaqMan™ Gene Expression Master Mix (Applied Biosystems/Thermo Fisher Scientific, Waltham, MA, USA). For miRNA, qPCR reactions were performed using TaqMan™ Universal PCR Master Mix, no AmpErase™ UNG (Applied Biosystems/Thermo Fisher Scientific, Waltham, MA, USA). Samples were run on a ViiA 7 Real-Time PCR System (Applied Biosystems/Thermo Fisher Scientific, Waltham, MA, USA) in 386 well plates. Relative expression values were calculated using the ΔΔCT method with normalization to the housekeeping gene [60].

### 4.5. Western Blot

Protein lysates were obtained from homogenized tissues in either T-PER (Thermo Fisher Scientific, Waltham, MA, USA; Ref# 78510) or M-PER lysis buffer (Thermo Fisher Scientific, Waltham, MA, USA; Ref# 78501) with 1x Halt™ Protease Phosphatase Inhibitor Cocktail (Thermo Fisher Scientific, Waltham, MA, USA; Ref# 78446). Protein lysates were quantified using a colorimetric Pierce BCA Protein Assay Kit (Thermo Fisher Scientific, Waltham, MA, USA; Ref# 23225). Whole protein lysates were used for the immunoblots and electrophoretically resolved on 4–20% Mini-PROTEAN^®^ TGX™ Precast Protein Gels (Bio-Rad Laboratories, Hercules, CA, USA; Ref# 4561094) or 10% Mini-PROTEAN^®^ TGX™ Precast Protein Gels (Bio-Rad Laboratories, Inc., Hercules, CA, USA; Ref# 4561034). Protein samples were then transferred to nitrocellulose membranes, and blocked in EveryBlot Blocking Buffer (Bio-Rad Laboratories, Inc., Hercules, CA, USA; Ref# 12010020). Primary antibody using Smad1/5/8 (Santa Cruz Biotechnology, Inc. Dallas, TX, USA; Ref# sc-6031, dilution = 1:500), P-Smad1/5/8 (MilliporeSigma, Burlington, MA, USA; Ref# AB3848-1, dilution = 1:200), Smad2/3 (Cell Signaling Technology, Inc., Danvers, MA, USA; Ref# 8685T, dilution = 1:500), P-Smad2 S465/467 (Cell Signaling Technology, Inc., Danvers, MA, USA; Ref# 3108T, dilution = 1:1,000), vinculin (MilliporeSigma, Burlington, MA, USA; Ref# V9131, dilution = 1:2,000) or gapdh were incubated overnight. Blots were washed and incubated with a secondary antibody conjugated to HRP with Precision Protein StrepTactin-HRP Conjugate (Bio-Rad Laboratories, Inc., Hercules, CA, USA; Ref#1610380) for 1 hour at room temperature. SuperSignal West Dura Extended Duration Substrate was used for enhanced chemiluminescent detection of protein bands (Thermo Fisher Scientific, Waltham, MA, USA; Ref# 34075). Blots were imaged using a BIO-RAD ChemiDoc MP system (Bio-Rad Laboratories, Inc., Hercules, CA, USA). The membranes were then stripped using Restore Plus Western Blot Stripping Buffer (Thermo Fisher Scientific, Waltham, MA, USA; Ref# 46428) and probed with a control primary antibody. Protein quantification was performed using ImageLab (Bio-Rad Laboratories, Inc., Version 6.10, Hercules, CA, USA) to obtain relative densitometry. Relative units were calculated by normalizing within blots to control lanes and then across blots relative to loading controls, either gapdh or vinculin. 

### 4.6. Myotube Fusion Index and Immunocytofluorescence Imaging

C2C12 cells were transfected with siSmad8 or siGFP (siControl) (Horizon Discovery Biosciences Limited, Cambridge, UK) using Lipofectamine 2000 in 6 well plates with coverslips placed in each well. Transfections were completed in duplicate to allow for qPCR analysis of *Smad8* mRNA and immunocytofluorescence (ICC). Cells were transfected and allowed to grow in GM for 48 hours before switching to DM. Cells were then allowed to differentiate for 4 days before fixation. For ICC, cells were fixed with 4% paraformaldehyde for 10 min at 4 °C with gentile agitation. Following fixation, the cells were washed twice in 1× phosphate-buffered saline (PBS), three times in 0.1% Triton-X/1 × PBS, and then incubated in blocking buffer (10% horse serum; 0.1% Triton-X; mixed in 1× PBS; Thermo Fisher Scientific, Waltham, MA, USA) for 1 h at room temperature with light shaking. Following blocking, the cells were incubated with MF20 primary antibody (Developmental Studies Hybridoma Bank, Iowa City, IA, USA; MF20, dilution = 1:200) overnight at 4 °C with gentle shaking. Cells were incubated in a secondary antibody, AlexaFluor-488 (Invitrogen/Thermo Fisher Scientific, Waltham, MA, USA) conjugated to mouse IgG. Cells were then incubated with 4′,6-diamidino-2-phenylindole and mounted with Vectashield mounting medium (Vector Laboratories, Inc., Newark, CA, USA). Cells that had been stained were imaged with Nikon Eclipse Ti2 inverted fluorescent microscope. Cells were imaged in 4 random fields. Greater than 350 nuclei were counter per group. Myotube fusion index was calculated as the fraction of myonuclei in MF20+ cells/myotubes out of the total number of myonuclei. The ratio was then normalized to the siControl group.

### 4.7. Model and Statistics

Models of Smad8 dysregulation in DMD were created using Biorender. Statistical analyses for human tissue and mouse data were performed in GraphPad Prism (GraphPad Software LLC., Version 9.3.1, San Diego, USA). A *t*-test was used to assess differences between control and disease groups. The level of statistical significance was set at 0.05 (two-sided). qPCR data were first normalized to reference genes (human and mouse total RNA: GAPDH, mouse miRNA: RNU6, human miRNA: RNU48) followed by normalization to control groups using the ΔΔCT method. 2-way ANOVA with Tukey’s multiple comparisons was used for statistical analysis of Figure 1A,B and Figure 6A,B. 6 outliers were identified and removed using the ROUT method in Figure 1B,C; this did not alter the statistically significant difference between the two groups. Bar graphs show individual biological replicates for each data point.

## 5. Conclusions

In summary, our findings show that Smad8 is increased in DMD muscle and may promote dystrophic signaling through its suppression of key myomiRs, inhibition of myoblast differentiation, and attenuation of disease-mitigating gene programs. We further show that BMP4, a signaling factor that is upregulated in DMD, induces and activates Smad8 in muscle cells and may represent a key driver of this signaling pathway in DMD. These findings present an interesting maladaptive role of Smad8 in repressing myomiR expression and suggest that inhibiting the BMP4/Smad8 pathway could have beneficial effects in DMD that are under-unexplored. Future studies assessing the impact of inhibiting the BMP4/Smad8 pathway will be helpful in elucidating potentially novel therapeutic targets in DMD.

## Data Availability

Data for this research is contained within the article.

## Supplementary Materials

The following supporting information can be downloaded at: https://www.mdpi.com/article/10.3390/ijms23147515/s1.
