# Supplementary material for: Smad8 Is Increased in Duchenne Muscular Dystrophy and Suppresses miR-1, miR-133a, and miR-133b
Source: Int J Mol Sci. 2022 Jul 7;23(14):7515. doi: 10.3390/ijms23147515 (PMC9323105; doi:10.3390/ijms23147515)
Supplement: Supplementary file 1 [file ijms-23-07515-s001.zip › ijms-1664076-supplementary.pdf]

Supplementary Materials

A

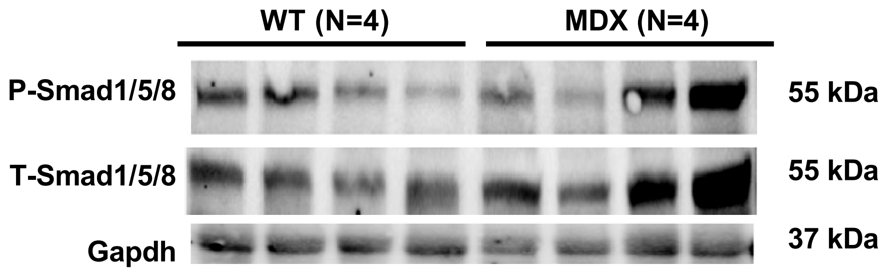

B

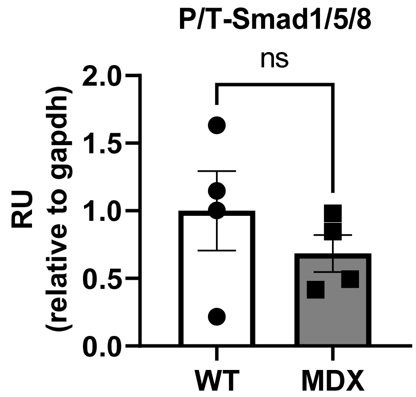

Figure S1. Smad1/5/8 total (T-Smad1/5/8) and phosphorylated (P-Smad1/5/8) protein in 6-month-old *mdx^{5cv}* tibialis anterior muscle. (A) Western blot showing T-Smad1/5/8, P-Smad1/5/8, and vinculin. (B) P/T-Smad1/5/8 ratio by densitometry. Data points show biological replicates for each group. Bars show means ± SEM. ns, not significant. RU, relative units.

Table S1 - Taqman Assays for Total RNA

Gene	Assay ID	Species
BMP4	hs03676628_s1	Human
GAPDH	hs02758991_g1	Human
ID1	hs03676575_s1	Human
IL-6	hs00174131_m1	Human
MEF2D	hs00954735_m1	Human
MYOG	hs01072232_m1	Human
SMAD1	hs00195432_m1	Human
SMAD2	hs00998187_m1	Human
SMAD3	hs00969210_m1	Human
SMAD5	hs00195437_m1	Human
SMAD9	hs00931723_m1	Human
TGFβ1	hs00998133_m1	Human
Gapdh	mm99999915_g1	Mouse
Id1	mm00775963	Mouse
Il-6	mm00435493	Mouse
Mef2d	mm00504931	Mouse
Myod	mm00440387	Mouse
Myog	mm00446194	Mouse
Nfkb1	mm00476361	Mouse
Smad2	mm00487530_m1	Mouse
Smad3	mm01170760_m1	Mouse
Smad9	mm00649885_m1	Mouse

Table S2 - Taqman assays for miRNA

miRNA	Stem / miRBase ID	Assay ID	Catalog No.	Species
miR-1	hsa-miR-1	002222	PN4427975	Human
miR-1	mmu-miR-1a-3p	002222	PN4427975	Mouse
miR-133a	hsa-miR-133a	002222	PN4427975	Human
miR-133a	mmu-miR-133a-3p	002222	PN4427975	Mouse
miR-133b	hsa-miR-133b	002222	PN4427975	Human
miR-133b	mmu-miR-133b-3p	002222	PN4427975	Mouse
pri-miR-1	mmu-mir-1a-2, mmu-mir-1b	mm03308741_pri	4427012	Mouse
pri-miR-133a	mmu-mir-133a-1	mm03306281_pri	4427012	Mouse
pri-miR-133b	mmu-mir-133b	mm03307410_pri	4427012	Mouse
RNU48	568908	001006	PN4427975	Human
RNU6	715680	001973	PN4427975	Mouse
